# O.4.3-10 Validation and investigation of a video-assisted version of the KIDSCREEN-27 for Danish children

**DOI:** 10.1093/eurpub/ckad133.196

**Published:** 2023-09-11

**Authors:** Mette Kurtzhals, Paulina Sander Melby, Glen Nielsen

**Affiliations:** Center for Clinical Research and Prevention, Copenhagen University Hospital – Bispebjerg and Frederiksberg, Nordre Fasanvej 57, 2000 Frederiksberg, Denmark; Department of Nutrition, Exercise and Sports, University of Copenhagen, Copenhagen N, Denmark; Department of Sports Science and Clinical Biomechanics, University of Southern Denmark, Campusvej 55, 5230 Odense, Denmark; mette.lindholm.kurtzhals@regionh.dk; Center for Clinical Research and Prevention, Copenhagen University Hospital – Bispebjerg and Frederiksberg, Nordre Fasanvej 57, 2000 Frederiksberg, Denmark; Department of Nutrition, Exercise and Sports, University of Copenhagen, Copenhagen N, Denmark

## Abstract

**Background:**

Poor well-being among children is an increasing concern worldwide. In Denmark, the decrease in well-being among children and youth has been evident in recent years making research on the promotion of well-being important. Three generic and comprehensive questionnaire-based measures of children’s well-being and quality of life of different lengths have been developed across 13 European countries (KIDSCREEN-52, KIDSCREEN-27, and KIDSCREEN-10). The questionnaires have been translated and validated in several languages – and the cross-cultural comparability and psychometric properties have been found satisfactory. However due to the required reading abilities for understanding and filling in the questionnaire the children’s self-report version has mainly been validated and used in ages 11-18 years. For this reason, parent proxy reporting is often used for children below age 11. In Denmark, the questionnaires have been translated, but no validation study has been published.

**Purpose:**

To test the measurement properties and evaluate the feasibility validity and reliability of a video-assisted version of the Danish KIDSCREEN 27 child self-report on a sample of Danish children aged six to nine years.

**Methods:**

An electronic version with video and speech-assisted instructions and explanations for the questionnaire was developed. The questionnaire was administered to a large sample of Danish school children aged six to nine years.

**Expected results and outcomes:**

We aim to improve the measurement of self-reported well-being among children aged six to nine years by using our video and speech-assisted electronic version of the KIDSREEN-27. Preliminary findings will be presented and discussed. The study is important, as its evident that we need a better understanding of the prerequisites, causes, pathways, and indicators of children’s well-being.

